# pH response and mechanical properties of Fe_2_O_3_–TeO_2_-based glass/stainless steel enamel electrodes for pH sensors

**DOI:** 10.1016/j.heliyon.2023.e12966

**Published:** 2023-01-18

**Authors:** Tadanori Hashimoto, Tomonari Kuno, Daiki Ito, Atsushi Ishihara, Yuji Nishio

**Affiliations:** a Division of Chemistry for Materials, Graduate School of Engineering, Mie University, 1577 Kurimamachiya-Cho, Tsu, Mie, 514-8507, Japan; b HORIBA Advanced Techno, Co., Ltd., 2 Miyanohigasi, Kisshoin, Minami-Ku, Kyoto, 601-8551, Japan

**Keywords:** Mechanical property, Three-point bending strength, Fe_2_O_3_–TeO_2_-based glass, Glass/stainless steel, Working electrode, pH sensor

## Abstract

Glass pH sensors are unsuitable for in vivo biomedical, clinical, or food applications because of the brittleness of glass and the difficulty in measuring small volumes. Enamel structures such as glass/stainless steel are candidates for glass-based pH electrodes. In this study, new enamel electrodes for pH sensors using Fe_2_O_3_–TeO_2_-based glass/stainless steel were developed. The effect of NiO addition to Fe_2_O_3_–TeO_2_ glass on the pH sensitivity and the three-point bending strength of enamels were investigated. The effect of NiO addition to Fe_2_O_3_–TeO_2_ glass/stainless steel on the pH sensitivity was negligible. Fe_2_O_3_–TeO_2_-based glass/stainless steel showed pH sensitivity appropriate to a working electrode. Enameling at a lower temperature under an air atmosphere was desirable for narrowing the gap between pH 4–7 and pH 7–9 sensitivities. The NiO addition to Fe_2_O_3_–TeO_2_ glass/stainless steel decreased the three-point bending strength. Therefore, NiO did not serve as an adhesion oxide in the Fe_2_O_3_–TeO_2_ glass. Fe_2_O_3_–TeO_2_ glass/stainless steel possessed the highest three-point bending strength among all samples when prepared at 670 °C under an air atmosphere. Therefore, no NiO addition and enameling at a lower temperature under an air atmosphere are desirable for obtaining more robust Fe_2_O_3_–TeO_2_ glass/stainless steel than Li_2_O–SiO_2_-based glass electrodes for pH sensors.

## 1 Introduction

Commercially available pH combination electrodes often consist of the following electrodes: (1) a working electrode that generates an electromotive force in response to the concentration of hydrogen ions in the solution and (2) a reference electrode. We have developed several working electrodes [1–7]. Recently, Ag and Ag alloy-precipitated Ag_2_O–TeO_2_ glass and Ag_2_O–TeO_2_ glass/stainless steel enamel reference electrodes for pH sensors were developed [8].

Glass pH sensors are unsuitable for in vivo biomedical, clinical, or food applications [9]. For example, the CO_2_ concentration of greenhouse gas affects human proteome performance through pH in human fluids [10]. Accurate pH measurements with high sensitivity are desired. Therefore, low-cost electrodes [[11], [12], [13]], all-solid-state pH electrodes [[14], [15], [16], [17], [18], [19]], disposable pH electrodes [[19], [20], [21]], and wearable pH electrodes [17,22,23] have been developed. In contrast, enamels with glass/metal structures [15,[24], [25], [26]] may resolve the disadvantage of being brittle, and metal electrodes [6,7,[27], [28], [29]] have relatively low chemical durability. For that, the synthesis and characterization of new materials is a critical topic for several applications [23,[29], [30], [31], [32], [33]]. We selected SUS304 stainless steel as a substrate for enameling because of its ease of handling compared with carbon steel. The coefficient of thermal expansion (CTE) of stainless steel 304 is 18 × 10^−6^ K^−1^ [34,35]. The CTE of the commercially available pH-responsive glass is 10 × 10^−6^ K^−1^ and is smaller than that of stainless steel 304 [15,36]. The CTE of the Fe_2_O_3_–Bi_2_O_3_-based glasses that we developed may also be smaller than that of stainless steel 304, according to the general data of Bi_2_O_3_-based glasses (8–11 × 10^−6^ K^−1^) [37]. In contrast, tellurite glasses [38,39] have a large CTE of 12–18 × 10^−6^ K^−1^, and this range is relatively close to that of stainless steel 304. Furthermore, Fe_2_O_3_–TeO_2_-based glasses [[40], [41], [42]] have lower glass transition temperatures desirable for enameling than those of Fe_2_O_3_–Bi_2_O_3_-based glasses [43].

The NiO and CoO are used as adhesion oxides between glass and stainless steel in typical enamels [[44], [45], [46], [47], [48]]. It has not been revealed whether NiO serves as an adhesion oxide in Fe_2_O_3_–NiO–TeO_2_ glass or not. Thus, Fe_2_O_3_–TeO_2_ glass-based glasses were selected as new glass/stainless steel enamels in the present study. Furthermore, the effect of NiO addition to Fe_2_O_3_–TeO_2_ enamels on the pH sensitivity and the three-point bending strength were investigated.

## 2 Experimental

### 2.1 Preparation of 20Fe*x*Ni(80-*x*)Te glasses

20Fe_2_O_3_･*x*NiO･(80-*x*)TeO_2_ (20Fe*x*Ni(80-*x*)Te, *x* = 0 and 10 mol%) glasses were produced via a conventional melt-quenching method. 20Fe_2_O_3_･*x*NiO･(80-*x*)TeO_2_ glass was abbreviated as 20Fe*x*Ni(80-*x*)Te. The following reagents were used as received: Fe_2_O_3_ (99.9% up, Kojundo Chemical Lab. Co., Ltd., Sakado, Japan), NiO (99.95% up, Kojundo Chemical Lab. Co., Ltd., Sakado, Japan) and TeO_2_ (99.9%, Kojundo Chemical Lab. Co., Ltd., Sakado, Japan). Batches (30 g) in alumina crucibles with caps were directly heat-treated at 900 °C for 1 h (20Fe80Te) and 1000 °C for 2 h (20Fe10Ni70Te) without mixing the melts. The melts were pressed via stainless steel, heat-treated at 300 °C, and annealed at 300 °C for 1 h 20Fe*x*Ni(80-*x*)Te glasses were polished for pH and electrical resistivity measurements.

### 2.2 Preparation of 20Fe*x*Ni(80-*x*)Te glass/stainless steel enamels

The 20Fe*x*Ni(80-*x*)Te glasses were crushed and classified as 20Fe*x*Ni(80-*x*)Te glass powder of less than 53 μm. The 20Fe*x*Ni(80-*x*)Te glass powder with a thickness of 1.0 mm was deposited on SUS304 stainless steel (Nilaco Corporation, Tokyo, Japan) with a thickness of 0.2 mm using the dry-type doctor blade method. Then, 20Fe*x*Ni(80-*x*)Te glass/stainless steel enamels (abbreviated 20Fe*x*Ni(80-*x*)Te/SUS) were obtained via heat-treatment at 670–730 °C for 0.5 h at the programming rate of 100 °C/min with (N_2_) and without (air) an N_2_ flow rate of 0.5 L/min, and then furnace cooling to fuse the 20Fe*x*Ni(80-*x*)Te glass powder onto the stainless steel. The stainless steel is a back electrode that supports the 20Fe*x*Ni(80-*x*)Te glasses at high heat-treatment temperatures because the stainless steel does not contact the test solution in potentiometric measurements.

### 2.3 Sample potentiometric measurements

**Fig. 1:**
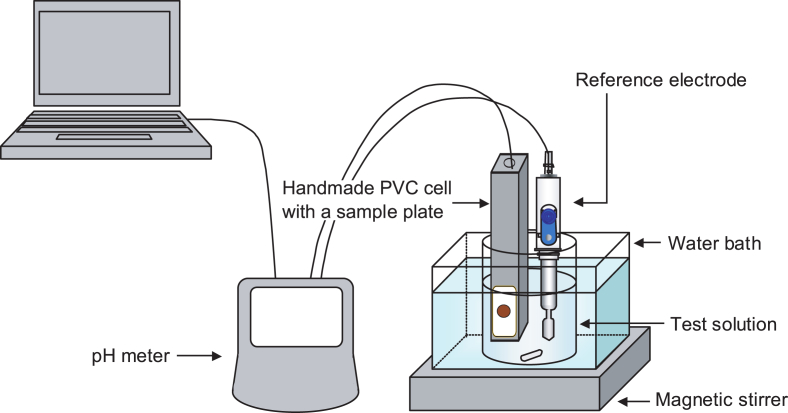
Apparatus for potentiometric measurement using a handmade PVC cell with a sample plate versus a commercially available reference electrode.

Potentiometric measurements for the 20Fe*x*Ni(80-*x*)Te glasses and 20Fe*x*Ni(80-*x*)Te/SUS were conducted at 25 °C using a handmade polyvinyl chloride (PVC) cell with a sample plate with dimensions of 16 mm × 16 mm × ∼1 mm (Fig. 1) and with an F-72 or F-74 pH meter (HORIBA, Ltd., Kyoto, Japan). The details of the pH measurement and pH responsivity (pH sensitivity, pH repeatability, and pH response time) were described in Refs. [[2], [3], [4], [5], [6], [7]].

### 2.4 Three-point bending strength measurement of samples

The mechanical properties of 20Fe*x*Ni(80-*x*)Te glasses and 20Fe*x*Ni(80-*x*)Te/SUS were measured using a ZTA-500 N digital force gauge (Imada Co., Ltd., Toyohashi, Japan). The BT-500 N three-point bending test fixture was set to a distance between the fulcrums of 10 mm (Imada Co., Ltd., Toyohashi, Japan). The MX2-1000-FA standard vertical motorized test stand was set to a cross-head speed of 2.5 mm/min (Imada Co., Ltd., Toyohashi, Japan) to evaluate the three-point bending strength.

### 2.5 Characterization of samples

The DC electrical resistivities of the 20Fe*x*Ni(80-*x*)Te glasses and 20Fe*x*Ni(80-*x*)Te/SUS with ∼1 mm thickness and an Ag electrode of 6 mm φ on both sides were measured at 25 °C using an SM-8215 super megohmmeter (HIOKI E. E. Corp., Ueda, Japan). Differential thermal analysis (DTA) measurements of 20Fe*x*Ni(80-*x*)Te glasses using DTG-60AH (Shimadzu Corp., Kyoto, Japan) were performed under air and N_2_ atmospheres. XRD patterns of the crystalline phases that precipitated in the 20Fe*x*Ni(80-*x*)Te glasses and 20Fe*x*Ni(80-*x*)Te/SUS were measured using an Ultima IV XRD measurement system (Rigaku Corp., Tokyo, Japan). The details of the DC electrical resistivity, DTA, and XRD measurements were described in Refs. [2–7].

## 3 Results and discussion

### 3.1 Thermal properties of 20Fe*x*Ni(80-*x*)Te glasses

The results of the DTA measurement of 20Fe*x*Ni(80-*x*)Te glasses are summarized in Table 1. The glass transition temperature (*T*_g_), crystallization peak temperature (*T*_p_), and *T*_p_-*T*_g_ related to the thermal stability of the glasses increased via NiO addition, whereas the melting temperature (*T*_m_) decreased. The *T*_g_ and *T*_p_ measured under an air atmosphere were higher than those measured under an N_2_ atmosphere. The transformation of Fe^2+^ into Fe^3+^ occurred under an air atmosphere. As a result, the viscosity of the glass increased, and crystallization became difficult [[49], [50], [51]]. This change in the valence of Fe ions under an air atmosphere is consistent with our XRD data, where the precipitation of Fe_2_O_3_ is suppressed, as will be seen in Section 3.2.2.

**Table 1:** Glass transition temperature (*T*_g_), crystallization peak temperature (*T*_p_), *T*_p_-*T*_g_, and melting temperature (*T*_m_) of 20Fe*x*Ni(80-*x*)Te glasses.

Sample Name	T_g_	T_p_	T_p_-T_g_	T_m_
	(°C)	(°C)	(°C)	(°C)
20Fe80Te (air)	417	533	116	648
20Fe80Te (N_2_)	406	525	119	648
20Fe10Ni70Te (air)	422	603	181	643
20Fe10Ni70Te (N_2_)	418	588	170	641

### 3.2 pH response of 20Fe*x*Ni(80-*x*)Te Glass/SUS

**Fig. 2:**
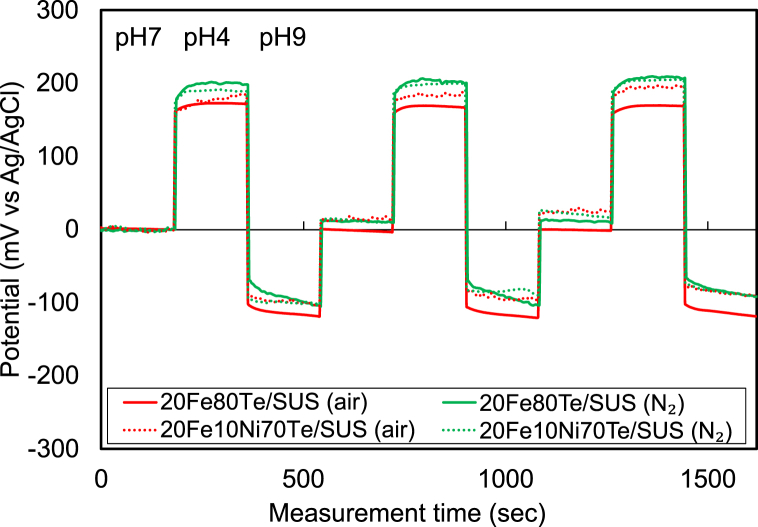
Changes in potential with the measurement time in pH 7, pH 4, and pH 9 buffer solutions for 20Fe*x*Ni(80-*x*)Te glass/SUS prepared at 730 °C.

Fig. 2 presents the changes in potential with the measurement time in pH 7, pH 4, and pH 9 buffer solutions for 20Fe*x*Ni(80-*x*)Te glass/SUS prepared at 730 °C. The change in potential with the measurement time corresponding to a pH change is significant for all samples, suggesting that it serves as a working electrode. The effect of the NiO addition and atmosphere for preparing glass/stainless steel enamels on pH response seems negligibly small.

**Fig. 3:**
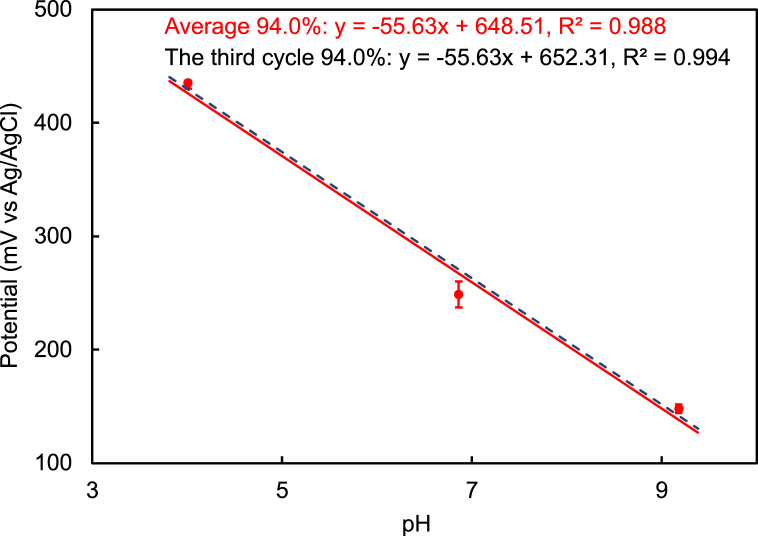
Relationship between potential and pH (pH 4, pH 7, and pH 9) for 20Fe80Te glass/SUS (670 °C, air) as a sample determined by the potential curve.

Fig. 3 shows the relationship between potential and pH (pH 4, pH 7, and pH 9) for 20Fe80Te glass/SUS (670 °C, air) as an example determined by the potential curve. The solid line is average data for three cycles, and the broken line is data for the third cycle. Good linear relationships with high coefficients of determination were observed for both lines. pH sensitivity of 94.0% was obtained from the relationship for 20Fe80Te glass/SUS (670 °C, air).

The pH responsivity (pH sensitivity, pH repeatability, and pH response time) of 20Fe*x*Ni(80-*x*)Te glasses and 20Fe*x*Ni(80-*x*)Te glass/SUS are listed in Table 2. Under “pH sensitivity”, the column entitled pH 4–9 presents the pH sensitivity between pH 4 and 9. The pH 4–7 sensitivity was higher than the pH 7–9 sensitivity for all samples. 20Fe*x*Ni(80-*x*)Te glasses showed lower pH 7–9 sensitivity than 20Fe*x*Ni(80-*x*)Te glass/SUS, but a difference occurred between pH 4–7 sensitivity and pH 7–9 sensitivity. The pH 4–9 sensitivity for 20Fe80Te glass/SUS (670 °C, air) in Table 2 was slightly different from that in Fig. 3 because two pHs determine the former, and three pHs determine the latter. The appropriate pH measurement of 20Fe*x*Ni(80-*x*)Te glasses and 20Fe*x*Ni(80-*x*)Te glass/SUS was conducted [8] because the electrical resistivity is less than 10^10^ Ω･cm, and a high pH sensitivity was obtained. The effect of the NiO addition on pH sensitivity was negligible for both 20Fe*x*Ni(80-*x*)Te glasses and 20Fe*x*Ni(80-*x*)Te glass/SUS. Enameling at a lower temperature under an air atmosphere was desirable for narrowing the gap between pH 4–7 and pH 7–9 sensitivities.

**Table 2:** pH responsivity (pH sensitivity, pH repeatability, and pH response time) of 20Fe*x*Ni(80-*x*)Te glasses and 20Fe*x*Ni(80-*x*)Te glass/SUS.

Sample Name	pH Sensitivity (%)			pH Repeatability (pH)/pH Response Time (sec)
	pH 4–7	pH 7–9	pH 4–9	
20Fe80Te/SUS (670 °C, air)	104.9	79.8	93.6	0.25/17
20Fe80Te/SUS (730 °C, air)	101.5	85.5	94.4	0.06/15
20Fe80Te/SUS (730 °C, N_2_)	117.6	73.2	97.6	0.16/20
20Fe80Te	90.2	82.1	86.6	0.38/4
20Fe10Ni70Te/SUS (730 °C, air)	102.1	85.0	94.5	0.42/13
20Fe10Ni70Te/SUS (730 °C, N_2_)	112.0	79.1	97.2	0.28/15
20Fe10Ni70Te	97.5	82.8	90.9	0.13/11

### 3.3 Three-point bending strength of samples

**Fig. 4:**
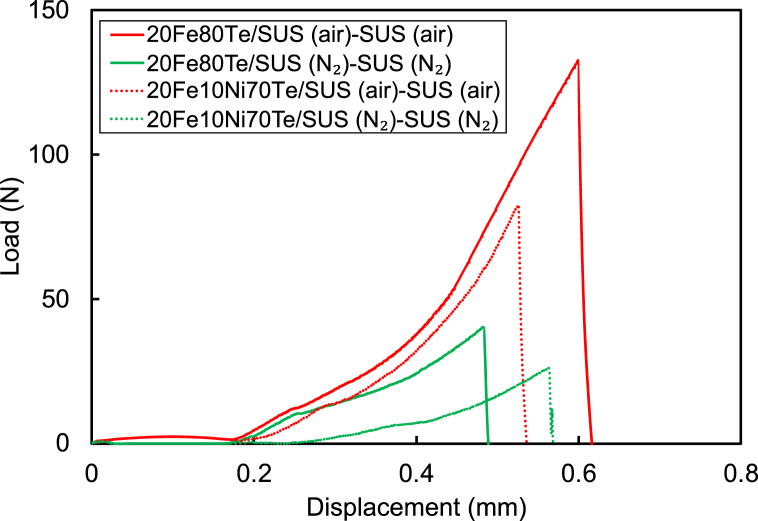
Corrected load-displacement curves for determining the three-point bending strength of 20Fe*x*Ni(80-*x*)Te glass/SUS prepared at 730 °C.

The corrected load-displacement curves for determining the three-point bending strength of 20FexNi(80-x)Te glass/SUS prepared at 730 °C are given in Fig. 4. In this figure, the curve of stainless steel heat-treated under the same atmosphere was subtracted from that of 20Fe*x*Ni(80-*x*)Te/SUS. The maximum load in these curves was defined as the three-point bending strength. Glass/stainless steel enamels heat-treated under air showed higher three-point bending strength than that heat-treated under N_2_.

Table 3 lists the three-point bending strength of 20Fe*x*Ni(80-*x*)Te/SUS and related samples. The NiO addition to Fe_2_O_3_–TeO_2_ glass/SUS decreased the three-point bending strength. Therefore, NiO did not serve as an adhesion oxide in the Fe_2_O_3_–NiO–TeO_2_ glass system. In contrast, the glass/SUS prepared under an air atmosphere showed higher three-point bending strength than those prepared under an N_2_ atmosphere. The effect was significant in 20Fe80Te/SUS (air). In addition, 20Fe80Te/SUS (air) possessed the highest three-point bending strength among all samples when prepared at 670 °C. The load-displacement curve for bare stainless steel did not show yield points and similar curves despite the atmosphere used. Therefore, the upper crystallized glass layers dominate the three-point bending strength of the glass/SUS. The effect of the precipitated crystals on the three-point bending strength is discussed in the following section.

**Table 3:** Three-point bending strength of 20Fe*x*Ni(80-*x*)Te glass/SUS and related samples.

Sample Name	Load (N)	Thickness (mm)
20Fe80Te/SUS (630 °C, air)	76.1 ± 11.9	0.775 ± 0.018
20Fe80Te/SUS (650 °C, air)	338.0 ± 43.4	0.823 ± 0.049
20Fe80Te/SUS (670 °C, air)	340.4 ± 36.2	0.735 ± 0.024
20Fe80Te/SUS (730 °C, air)	179.5 ± 27.2	0.499 ± 0.029
20Fe80Te/SUS (730 °C, N_2_)	62.4 ± 23.6	0.524 ± 0.019
20Fe10Ni70Te/SUS (730 °C, air)	85.4 ± 21.2	0.732 ± 0.068
20Fe10Ni70Te/SUS (730 °C, N_2_)	54.2 ± 6.3	0.685 ± 0.043
20Fe80Te	19.6 ± 10.7	0.706 ± 0.028
20Fe10Ni70Te	14.5 ± 4.7	0.712 ± 0.016
HORIBA	37.8 ± 3.0	0.581 ± 0.019

### 3.4 Crystals precipitated in 20Fe*x*Ni(80-*x*)Te Glass/SUS

#### 3.4.1 Effect of NiO addition on three-point bending strength

**Fig. 5:**
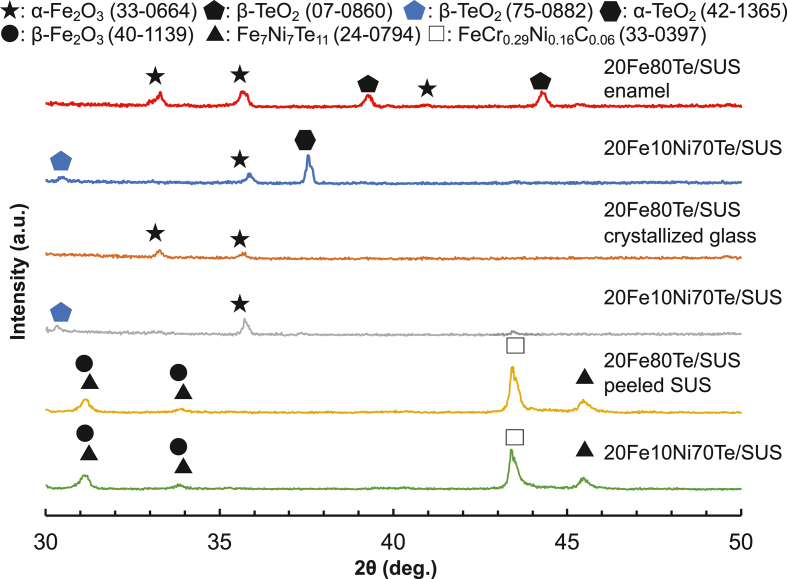
XRD patterns of (a) 20Fe*x*Ni(80-*x*)Te glass/SUS (730 °C, air) before the three-point bending strength measurement, (b) the crystallized glass separated from enamel after the three-point bending strength measurement, (c) the stainless steel peeled off enamel after the three-point bending strength measurement.

Fig. 5 shows the XRD patterns of (a) 20Fe*x*Ni(80-*x*)Te glass/SUS (730 °C, air) before the three-point bending strength measurement, (b) the crystallized glass separated from enamel after the three-point bending strength measurement, and (c) the stainless steel peeled off enamel after the three-point bending strength measurement. α-Fe_2_O_3_ (JCPDS card No. 33–0664) and β-TeO_2_ (JCPDS card No. 07–0860) for 20Fe80Te/SUS and α-Fe_2_O_3_ and α-TeO_2_ (JCPDS card No. 42–1365), and β-TeO_2_ (JCPDS card No. 75–0882) for 20Fe10Ni70Te/SUS were observed on the surface of the 20Fe*x*Ni(80-*x*)Te glass/SUS. α-Fe_2_O_3_ for 20Fe80Te/SUS and α-Fe_2_O_3_ and β-TeO_2_ (JCPDS card No. 75–0882) for 20Fe10Ni70Te/SUS were observed in the interior of the crystallized glass. FeCr_0.29_Ni_0.16_C_0.06_ (JCPDS card No. 33–0397), β-Fe_2_O_3_ (JCPDS card No. 40–1139), and Fe_7_Ni_7_Te_11_ (JCPDS card No. 24–0794) were observed at the crystallized glass and stainless steel interface for both samples. FeCr_0.29_Ni_0.16_C_0.06_ is after SUS304 as a substrate. β-Fe_2_O_3_ and Fe_7_Ni_7_Te_11_ were formed after enameling.

The three-point bending strength of 20Fe10Ni70Te/SUS (730 °C, air) was half that of 20Fe80Te/SUS (730 °C, air). The diffraction line of α-TeO_2_ (JCPDS card No. 42–1365) observed on the surface of 20Fe10Ni70Te/SUS (730 °C, air) was prominent. Therefore, α-TeO_2_ may be the starting point of destruction and decrease the three-point bending strength. A significant difference in the crystallized glass and the peeled stainless steel was not seen in the present case.

#### 3.4.2 Effect of enamel preparation atmosphere on three-point bending strength

**Fig. 6:**
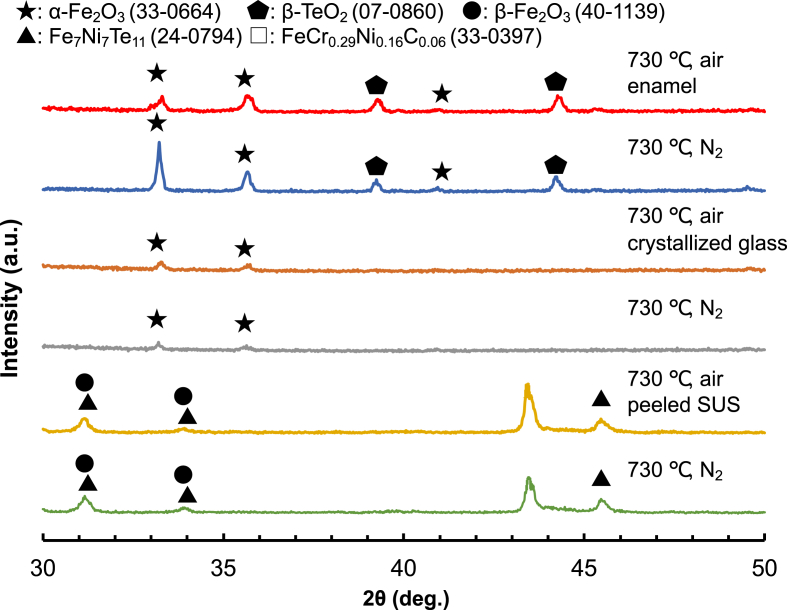
XRD patterns of (a) 20Fe80Te glass/SUS (730 °C, air and N_2_) before the three-point bending strength measurement, (b) the crystallized glass separated from enamel after the three-point bending strength measurement, and (c) the stainless steel peeled off enamel after the three-point bending strength measurement.

The XRD patterns of (a) 20Fe80Te glass/SUS (730 °C, air and N_2_) before the three-point bending strength measurement, (b) the crystallized glass separated from enamel after the three-point bending strength measurement, and (c) the stainless steel peeled off enamel after the three-point bending strength measurement are represented in Fig. 6 α-Fe_2_O_3_ and β-TeO_2_ were observed on the surface of 20Fe80Te/SUS (730 °C, air and N_2_). α-Fe_2_O_3_ was observed in the interior of the crystallized glass for both samples. FeCr_0.29_Ni_0.16_C_0.06_, β-Fe_2_O_3_, and Fe_7_Ni_7_Te_11_ were observed at the crystallized glass and stainless steel interface for both samples.

The three-point bending strength of 20Fe80Te/SUS (N_2_) was one-third of that of 20Fe80Te/SUS (air). The peak intensity of α-Fe_2_O_3_ on the surface of 20Fe80Te/SUS (N_2_) was much higher than that of 20Fe80Te/SUS (air). It is well known that a decrease in Fe^3+^ decreases glass viscosity [49–51]. Therefore, glass fusion under a N_2_ atmosphere induces glass crystallization. Our results are consistent with previous reports. Therefore, α-Fe_2_O_3_ may be the starting point of destruction and decrease the three-point bending strength. A significant difference in the crystallized glass was not seen in the present case. The crystallinity of FeCr_0·29_Ni_0·16_C_0.06_ due to SUS304 was high for 20Fe80Te/SUS (air). However, the negative effect of FeCr_0·29_Ni_0·16_C_0.06_ on the three-point bending strength seems negligible.

#### 3.4.3 Effect of enamel preparation temperature on three-point bending strength

**Fig. 7:**
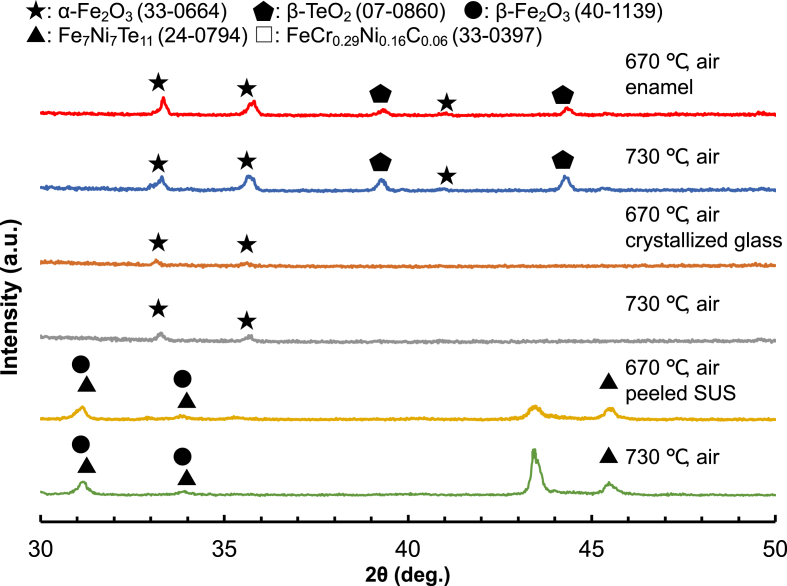
XRD patterns of (a) 20Fe80Te glass/SUS (670 °C, air, and 730 °C, air) before the three-point bending strength measurement, (b) the crystallized glass separated from enamel after the three-point bending strength measurement, and (c) the stainless steel peeled off enamel after the three-point bending strength measurement.

Fig. 7 shows the XRD patterns of (a) 20Fe80Te glass/SUS (670 °C, air, and 730 °C, air) before the three-point bending strength measurement, (b) the crystallized glass separated from enamel after the three-point bending strength measurement, and (c) the stainless steel peeled off enamel after the three-point bending strength measurement. α-Fe_2_O_3_ and β-TeO_2_ were observed on the surface of 20Fe80Te glass/SUS (670 °C, air, and 730 °C, air). α-Fe_2_O_3_ and β-TeO_2_ were observed in the interior of the crystallized glass for both samples. FeCr_0.29_Ni_0.16_C_0.06_, β-Fe_2_O_3_, and Fe_7_Ni_7_Te_11_ were observed at the crystallized glass and stainless steel interface for both samples.

The three-point bending strength of 20Fe80Te/SUS (730 °C, air) was half that of 20Fe80Te/SUS (670 °C, air). The peak intensity of β-TeO_2_ on the surface 20Fe80Te/SUS (730 °C, air) was higher than that of 20Fe80Te/SUS (670 °C, air). Therefore, β-TeO_2_ may be the starting point of destruction and decrease the three-point bending strength. A significant difference in the crystallized glass was not seen in the present case. The crystallinity of FeCr_0·29_Ni_0·16_C_0.06_ due to the SUS304 of 20Fe80Te/SUS (730 °C, air) was stronger than that of 20Fe80Te/SUS (670 °C, air). However, the effect of the enhanced crystallinity of stainless steel at the crystallized glass and stainless steel interface on the three-point bending strength seems negligible, as in the case of NiO addition (Fig. 4) and atmosphere control (Fig. 5).

On the other hand, an enameling temperature (630 °C) that was too low resulted in a low three-point bending strength because of low glass fusion. According to these results, no NiO addition and enameling at appropriate low enameling temperatures under an air atmosphere are desirable for obtaining robust enamels. These conditions suppressed the formation of α-Fe_2_O_3_ and α-TeO_2_ on the surface of enamels. Furthermore, 20Fe80Te/SUS (670 °C, air) was much more robust than commercially available Li_2_O–SiO_2_-based glass electrodes for pH sensors. Our result may show that Fe_2_O_3_-containing materials, such as novel ferrite nanoparticles [52], are candidates for new pH and other electrodes.

## 4 Conclusions

New enamel electrodes for pH sensors using Fe_2_O_3_–TeO_2_-based glass/SUS were developed in this study. The effect of NiO addition to Fe_2_O_3_–TeO_2_ glass/SUS on the pH sensitivity and the three-point bending strength were investigated.

● The effect of NiO addition to Fe_2_O_3_–TeO_2_ glass/SUS on pH sensitivity was negligible. Fe_2_O_3_–TeO_2_-based glass/SUS showed appropriate pH sensitivity as a working electrode. Enameling at a lower temperature under an air atmosphere was desirable for narrowing the gap between pH 4–7 and pH 7–9 sensitivities.
● The NiO addition to Fe_2_O_3_–TeO_2_ glass/SUS decreased the three-point bending strength. Therefore, NiO did not serve as an adhesion oxide in the Fe_2_O_3_–TeO_2_ glass. In contrast, Fe_2_O_3_–TeO_2_ glass/SUS possessed the highest three-point bending strength among all samples when prepared at 670 °C under an air atmosphere. These conditions suppressed the formation of α-Fe_2_O_3_ and α-TeO_2_ on the surface of enamels.
● No NiO addition or enameling at a lower temperature under an air atmosphere is desirable for obtaining robust enamels. 20Fe80Te/SUS (670 °C, air) was much more robust than commercially available Li_2_O–SiO_2_-based glass electrodes for pH sensors.

## CRediT author statement

Tadanori Hashimoto: Conceptualization, Methodology, Writing-Original draft preparation, Supervision, Funding acquisition. Tomonari Kuno: Investigation, Visualization. Daiki Ito: Investigation, Visualization. Atsushi Ishihara: Writing-Review & Editing, Yuji Nishio: Resources, Writing-Review & Editing.

## Funding statement

This work was supported by JSPS KAKENHI (JP18K04702 and JP22K04685).

## Declaration of competing interest

The authors declare that they have no known competing financial interests or personal relationships that could have appeared to influence the work reported in this paper.

## Abbreviations

20Fe*x*Ni(80-*x*)Te: 20Fe_2_O_3_･*x*NiO･(80-*x*)TeO_2_
20Fe80Te/SUS: 20Fe_2_O_3_･80TeO_2_ glass/stainless steel
20Fe10Ni70Te/SUS: 20Fe_2_O_3_･10NiO･70TeO_2_ glass/stainless steel
CTE: coefficient of thermal expansion
DTA: differential thermal analysis
*T*_g_: glass transition temperature
*T*_p_: crystallization peak temperature
*T*_m_: melting temperature
XRD: X-ray diffraction
